# Dietary patterns and survival in German postmenopausal breast cancer survivors

**DOI:** 10.1038/bjc.2012.521

**Published:** 2012-11-20

**Authors:** A Vrieling, K Buck, P Seibold, J Heinz, N Obi, D Flesch-Janys, J Chang-Claude

**Affiliations:** 1 Unit of Genetic Epidemiology, Division of Cancer Epidemiology, German Cancer Research Center (DKFZ), Im Neuenheimer Feld 581, 69120 Heidelberg, Germany; 2 Department of Cancer Epidemiology/Clinical Cancer Registry, University Cancer Center Hamburg (UCCH), Martinistrasse 52, 20246 Hamburg, Germany; 3 Department of Medical Biometry and Epidemiology, Center for Experimental Medicine, University Medical Center Hamburg-Eppendorf, Martinistrasse 52, 20246 Hamburg, Germany

**Keywords:** breast cancer, dietary pattern, mortality, recurrence

## Abstract

**Background:**

Research on the association between dietary patterns and breast cancer survival is very limited.

**Methods:**

A prospective follow-up study was conducted in Germany, including 2522 postmenopausal breast cancer patients diagnosed in 2001–2005 with available food frequency questionnaire data. Vital status, causes of death, and recurrences were verified through the end of 2009. Principle component factor analysis was used to identify pre-diagnostic dietary patterns. Hazard ratios (HRs) and 95% confidence intervals (CIs) were calculated with Cox proportional hazards models.

**Results:**

Two major dietary patterns were identified: ‘healthy’ (high intakes of vegetables, fruits, vegetable oil, sauces/condiments, and soups/bouillons) and ‘unhealthy’ (high intakes of red meat, processed meat, and deep-frying fat). Increasing consumption of an ‘unhealthy’ dietary pattern was associated with an increased risk of non-breast cancer mortality (highest *vs* lowest quartile: HR, 3.69; 95% CI, 1.66–8.17; *P-*trend <0.001). No associations with breast cancer-specific mortality and breast cancer recurrence were found. The ‘healthy’ dietary pattern was inversely associated with overall mortality (HR, 0.74; 95% CI, 0.47–1.15; *P-*trend=0.02) and breast cancer recurrence (HR, 0.71; 95% CI, 0.48–1.06; *P-*trend=0.02) in stage I–IIIa patients only.

**Conclusion:**

Increasing intake of an ‘unhealthy’ pre-diagnostic dietary pattern may increase the risk of non-breast cancer mortality, whereas increasing intake of a ‘healthy’ pattern may reduce the risk of overall mortality and breast cancer recurrence.

Two prospective cohort studies examined the association of dietary patterns with prognosis in breast cancer patients in the United States. In both studies, a lower pre- and post-diagnostic intake of the Western dietary pattern and a higher post-diagnostic intake of the prudent dietary pattern were associated with decreased mortality from causes unrelated to breast cancer (Kroenke *et al*, 2005; Kwan *et al*, 2009). No associations with breast cancer-specific mortality (Kroenke *et al*, 2005; Kwan *et al*, 2009) and breast cancer recurrence (Kwan *et al*, 2009) were found. Dietary patterns are different between countries, and data outside of the United States on dietary patterns and breast cancer prognosis are limited. Thus, we examined the association of pre-diagnostic dietary patterns with mortality and breast cancer recurrence in a large cohort of German postmenopausal breast cancer survivors.

## Materials and methods

### Study population

Patients were recruited in 2002–2005 within a large population-based case–control study on breast cancer in Hamburg and the Rhein-Neckar-Karlsruhe region in Germany (the MARIE study; Flesch-Janys *et al*, 2008), and followed up to the end of 2009. Patients had histologically confirmed primary invasive (stage I to IV) or *in situ* breast cancer, and were aged 50–74 years at diagnosis in 2001–2005. From 3464 postmenopausal patients, 2522 postmenopausal invasive breast cancer patients remained after excluding those with missing food frequency questionnaire (FFQ; *n*=520), previous cancer (*n*=207), *in situ* breast cancer (*n*=165), or energy intake in the bottom or top 1.0 percentile (*n*=50). This study was approved by the ethics committee of the University of Heidelberg, the ethical review board of Hamburg Medical Council, and the Medical Board of the State of Rheinland-Pfalz, and conducted in accordance with the Declaration of Helsinki.

### Data collection

Besides a face-to-face interview at recruitment, patients completed a validated, self-administered 176-item FFQ (Bohlscheid-Thomas *et al*, 1997a, 1997b), referring to the year before diagnosis and including 6 additional food items rich in phytoestrogens. The FFQ was comparable to the one used in the German part of the European Prospective investigation into Cancer and Nutrition study, with similar detailed written instructions and quality procedures. For each food item, the information about portion size and consumption frequency was used to calculate intakes in grams per day. Nutrients were estimated using the German food composition table BLS II.3 (Bundesinstitut für Gesundheitlichen Verbraucherschutz und Veterinärmedizin). The FFQ food items were assigned to one of 80 food classes using the EPIC-Soft software (Voss *et al*, 1998), and grouped into 25 subordinated food groups based on nutrient profiles or culinary usage according to the common classification of the EPIC project (Slimani *et al*, 2002; Supplementary Figure 1 and Table 1).

### Outcome assessment

Vital status of participants was determined through population registries up to the end of 2009. Causes of death were extracted from death certificates. Medical records were checked or treating physicians were contacted to identify recurrences and to verify self-reported events collected during a follow-up telephone interview conducted from May to September 2009 (>90% self-reported events verified). Recurrence included ipsilateral/contralateral/local/regional invasive recurrence and distant recurrence, and analyses for this endpoint were restricted to participants with stage I–IIIa disease, as well as information on recurrences occurring after recruitment into the study (*n*=2184). Participants without event of interest were censored at the date of last contact or 31 December 2009, whichever came first.

### Statistical analyses

Principle component factor analysis was used to extract factors explaining the maximum proportion of the variance in the correlation matrix of the 25 food groups, and performed using SAS software 9.2 (SAS Institute, Cary, NC, USA). The number of factors to be retained in the model was determined by eigenvalues of the correlation matrix (>1), Scree plots, and the natural interpretability of each factor. An orthogonal transformation (varimax) was used to rotate the correlation matrix to get a simpler data structure with greater interpretability (Rencher, 2002). Each individual was assigned a factor score for each identified pattern using the SAS procedure SCORE. Thus, individuals with high scores for a dietary pattern have a greater tendency to follow the pattern than individuals with a low score. Labelling of the factor (i.e., dietary pattern) was performed quantitatively using a cut-off value of 0.30 of the factor loadings.

Delayed-entry Cox proportional hazards models, based on time since recruitment until event/censoring, were used to examine the association of the factor score with survival and recurrence (Therneau and Grambsch, 2000). Hazard ratios (HRs) and 95% confidence intervals (CIs) were calculated using the factor score for dietary pattern as categorical variable divided into quartiles. The lowest quartile was defined as the reference category. All analyses were stratified by age at diagnosis and study centre. In multivariable models, we adjusted for tumour size, nodal status, primary metastasis, tumour grade, oestrogen/progesterone receptor status, total energy intake, and for all confounders that contributed significantly to the model or influenced the HRs more than 10% (radiotherapy, mode of detection, and hormone replacement therapy (HRT) use at diagnosis; for variable categorisation see Supplementary Table 1). Tests for linear trend with log HR were performed using the factor score for dietary pattern as a continuous variable. All tests were two-sided and considered to be statistically significant if *P*-value <0.05. All statistical analyses were performed using the SAS software 9.2 (SAS Institute).

## Results

Women were enroled in the study a median time of 97 days after diagnosis, and median follow-up time was 5.5 years. Overall, 316 deaths occurred, 235 due to breast cancer and 81 due to non-breast cancer causes (39 other cancers, 20 cardiovascular disease, and 22 other causes). Of the 2184 patients with stage I–IIIa disease and available data on recurrence status, 247 had a breast cancer recurrence.

Two major pre-diagnostic dietary patterns were defined: (1) high vegetables, fruits, vegetable oil, sauces/condiments, and soups/bouillons intake (‘healthy’ pattern), explaining 45.8% of the variance; and (2) high red meat, processed meat, and deep-frying fat intake (‘unhealthy’ pattern), explaining 29.8% of the variance (Table 1). Women in the highest compared with the lowest quartile of the ‘healthy’ pattern were more likely to be past smokers, more physically active, and had a higher educational and occupational level. For women in the highest quartile of the ‘unhealthy’ pattern, this was the other way around (Supplementary Table 1).

Women in the highest compared with the lowest quartile of the ‘unhealthy’ pattern were at an increased risk of overall mortality, driven by an increased risk of non-breast cancer mortality (HR, 3.69; 95% CI, 1.66–8.17; *P*-trend<0.001; Table 2). Results were similar but attenuated when restricted to stage I–IIIa patients (Supplementary Table 2). No associations were observed between the ‘unhealthy’ pattern and breast cancer-specific mortality (Table 2) or breast cancer recurrence (Table 3). The ‘healthy’ pattern was not associated with any of the mortality outcomes in the total population (Table 2), but in stage I–IIIa patients an inverse association with overall mortality (HR, 0.74; 95% CI, 0.47–1.15; *P-*trend=0.02) and breast cancer recurrence (HR, 0.71; 95% CI, 0.48–1.06; *P-*trend=0.02) was found (Supplementary Table 2 and Table 3). We observed no significant effect modification by BMI, HRT use, smoking status, leisure time physical activity, educational level, and ER status (data not shown).

## Discussion

In our study of German postmenopausal breast cancer patients, a higher pre-diagnostic intake of an ‘unhealthy’ dietary pattern was associated with an increased risk of non-breast cancer mortality, whereas the ‘healthy’ pattern was inversely associated with risk of overall mortality and breast cancer recurrence among stage I–IIIa patients only.

Similar results were found by two previous US studies for the mortality outcomes with either pre-diagnostic (Kroenke *et al*, 2005) or post-diagnostic dietary patterns (Kroenke *et al*, 2005; Kwan *et al*, 2009). However, a post-diagnostic ‘healthy’ pattern was not previously associated with a reduced breast cancer recurrence risk (Kwan *et al*, 2009). A randomised clinical trial of a diet high in vegetables, fruit and fibre, and low in fat, found no effect on breast cancer recurrence and mortality (Pierce *et al*, 2007). Therefore, our findings have to be confirmed by other studies. In disease-free women, an ‘unhealthy’ dietary pattern was also associated with increased risk of mortality from cardiovascular disease (Shimazu *et al*, 2007; Heidemann *et al*, 2008), whereas the opposite was true for a ‘healthy’ dietary pattern (Brunner *et al*, 2008; Heidemann *et al*, 2008). As postmenopausal breast cancer survivors frequently suffer from comorbid conditions (Patnaik *et al*, 2011), making healthier dietary choices may provide a means to improve their overall prognosis.

The dietary patterns we identified are somewhat different from those in the US studies. One explanation is that factor analysis is subject to several subjective decisions (Martinez *et al*, 1998). Further, we used a different FFQ in our study, resulting in a different classification into food groups. Also, we could not differentiate between low- and high-fat dairy, and between whole and refined grains, which are important characteristics of the prudent and Western pattern. Finally, eating habits are different between Germany and the United States.

Strengths of our study are the relatively large sample size, population-based design, and nearly complete follow-up. Limitations include the use of the FFQ. Although this is a validated dietary assessment methodology, non-captured dietary factors may have gone undetected, and recall bias and measurement errors cannot be ruled out. Also, potential changes in dietary patterns after diagnosis cannot be accounted for. We cannot exclude residual or uncontrolled confounding, and had a limited sample size for subgroup analyses.

In conclusion, higher intake of a pre-diagnostic ‘unhealthy’ dietary pattern may increase the risk of non-breast cancer mortality, whereas a higher intake of a ‘healthy’ dietary pattern may decrease the risk of overall mortality and breast cancer recurrence.

## Figures and Tables

**Table 1:** Food group factor loadings for the ‘healthy’ and ‘unhealthy’ dietary pattern

**Food group**	**‘Healthy’ pattern**	**‘Unhealthy’ pattern**
Potatoes	0.01	0.27
Vegetables	**0.71**	0.09
Legumes	0.15	0.07
Fruits	**0.37**	−0.11
Dairy products	0.10	−0.05
Pasta, rice, and other grain	0.25	−0.08
Bread	−0.04	0.23
Other cereals	0.15	−0.17
Red meat	0.12	**0.52**
Processed meat	−0.02	**0.52**
Poultry	0.19	0.29
Fish and shellfish	0.18	0.04
Eggs and egg products	−0.01	0.18
Vegetable oils	**0.62**	0.07
Butter	−0.15	0.18
Margarine	0.02	0.16
Deep-frying fat	0.01	**0.32**
Sugar and confectionery	−0.02	0.15
Cakes	−0.01	0.11
Non-alcoholic beverages	0.20	−0.03
Wine	0.05	−0.04
Other alcoholic beverages	−0.06	0.03
Sauces and condiments	**0.36**	0.16
Soups and bouillons	**0.30**	0.09
Soy products	0.09	−0.10

Factor loadings ⩾0.30/⩽−0.30 are shown in bold.

**Table 2:** HRs of overall mortality, breast cancer-specific mortality, and other mortality according to quartiles of dietary patterns in the MARIE study, Germany, 2001–2009

		**Overall mortality**			**Breast cancer mortality**			**Other mortality**		
**Quartiles of dietary pattern**	**No. of subjects**	**No. of deaths**	**HR**	**95% CI**	**No. of deaths**	**HR**	**95% CI**	**No. of deaths**	**HR**	**95% CI**
**‘Healthy’ pattern**										
**Model 1^a^**										
Q1	631	101	1.00		75	1.00		26	1.00	
Q2	630	68	0.71	0.52, 0.97	50	0.67	0.47, 0.96	18	0.84	0.45, 1.54
Q3	631	74	0.77	0.56, 1.04	57	0.76	0.54, 1.09	17	0.77	0.41, 1.45
Q4	630	73	0.77	0.56, 1.05	53	0.73	0.51, 1.05	20	0.89	0.49, 1.63
*P*-trend			0.06			0.04			0.80	
										
**Model 2^b^**										
Q1	614	97	1.00		72	1.00		25	1.00	
Q2	616	62	0.77	0.55, 1.08	46	0.76	0.51, 1.14	16	0.81	0.41, 1.57
Q3	617	72	0.81	0.58, 1.13	55	0.83	0.56, 1.23	17	0.82	0.42, 1.58
Q4	609	68	0.87	0.61, 1.23	50	0.89	0.59, 1.35	18	0.81	0.40, 1.61
*P*-trend			0.20			0.25			0.66	
										
**‘Unhealthy’ pattern**										
**Model 1^a^**										
Q1	631	72	1.00		61	1.00		11	1.00	
Q2	630	69	0.95	0.68, 1.32	48	0.77	0.52, 1.13	21	1.96	0.93, 4.12
Q3	631	76	1.06	0.76, 1.47	60	1.00	0.69, 1.43	16	1.42	0.65, 3.08
Q4	630	99	1.39	1.02. 1.89	66	1.06	0.74, 1.50	33	3.41	1.69, 6.85
*P*-trend			0.01			0.44			<0.001	
										
**Model 2^b^**										
Q1	619	69	1.00		59	1.00		10	1.00	
Q2	615	67	1.03	0.72, 1.47	46	0.88	0.58, 1.33	21	2.06	0.94, 4.54
Q3	611	72	1.11	0.77, 1.59	58	1.07	0.71, 1.61	14	1.46	0.63, 3.39
Q4	611	91	1.34	0.93, 1.94	60	0.99	0.64, 1.52	31	3.69	1.66, 8.17
*P*-trend			0.03			0.59			<0.001	

Abbreviations: CI=confidence interval; ERPR=oestrogen receptor/progesterone receptor; HR=hazard ratio; HRT=hormone replacement therapy; Q=quartile.

a The model was stratified by age at diagnosis and study centre.

b The model was stratified by age at diagnosis and study centre, and adjusted for tumour size, nodal status, metastases, tumour grade, ERPR status, radiotherapy, HRT use at diagnosis, mode of detection, and total energy intake; the model for other mortality was additionally adjusted for cardiovascular disease; because of missing covariate values, 66 observations were not included in model 2. Other potentially confounding variables, as specified in Supplementary Table 1, were not statistically significant and did not change the risk estimates by ⩾10% when tested in the model and were therefore not included in the final model.

**Table 3:** HRs of recurrence among stage I–IIIa breast cancer patients according to quartiles of dietary patterns in the MARIE study, Germany, 2001–2009

		**Recurrence**		
**Quartiles of dietary pattern**	**No. of subjects**	**No. of recurrences**	**HR**	**95% CI**
**‘Healthy’ pattern**				
**Model 1^a^**				
Q1	527	69	1.00	
Q2	554	58	0.82	0.58, 1.17
Q3	553	62	0.82	0.57, 1.16
Q4	550	58	0.84	0.59, 1.21
*P*-trend			0.11	
			
**Model 2^b^**				
Q1	517	67	1.00	
Q2	542	56	0.84	0.58, 1.21
Q3	543	61	0.77	0.54, 1.12
Q4	533	55	0.71	0.48, 1.06
*P*-trend			0.02	
				
**‘Unhealthy’ pattern**				
**Model 1^a^**				
Q1	557	60	1.00	
Q2	549	61	1.02	0.71, 1.46
Q3	539	52	0.88	0.60, 1.28
Q4	539	74	1.20	0.85, 1.70
*P*-trend			0.32	
			
**Model 2^b^**				
Q1	546	59	1.00	
Q2	538	60	0.99	0.68, 1.44
Q3	527	52	0.76	0.51, 1.13
Q4	524	68	0.91	0.61, 1.36
*P*-trend			0.72	

Abbreviations: CI=confidence interval; ERPR=oestrogen receptor/progesterone receptor; HR=hazard ratio; HRT=hormone replacement therapy; Q=quartile.

a The model was stratified by age at diagnosis and study centre.

b The model was stratified by age at diagnosis and study centre, and adjusted for tumour size, nodal status, metastases, tumour grade, ERPR status, radiotherapy, HRT use at diagnosis, mode of detection, and total energy intake; because of missing covariate values, 49 observations were not included in model 2. Other potentially confounding variables, as specified in Supplementary Table 1, were not statistically significant and did not change the risk estimates by ⩾10% when tested in the model and were therefore not included in the final model.
