# Supplementary material for: Dietary patterns and survival in German postmenopausal breast cancer survivors
Source: Br J Cancer. 2012 Nov 20;108(1):188–92. doi: 10.1038/bjc.2012.521 (PMC3553521; doi:10.1038/bjc.2012.521)
Supplement: Supplementary Figure 1 [file bjc2012521x1.doc]

**Supplementary Figure 1.** Definition of 25 food groups for principal component factor analysis based on slightly modified versions of the EPIC-SOFT food (sub-) groups defined by Slimani *et al*, 2002.

| 176 FFQ items |  |  |
| --- | --- | --- |
| ↓ |  |  |
| 80 food classes |  |  |
| ↓  ↓ | → | 4 ‘unclassified’ food classes |
|  | → | 3 other food classes (spices, herbs, and flavourings; chicory, substitutes; artificial sweeteners) |
| 73 food classes |  |  |
| ↓ |  |  |
| 12 main food groups + 12 subgroups + soy products group | | |
| ↓ |  |  |
| 1. Potatoes | | |
| 2. Vegetables | | |
| 3. Legumes | | |
| 4. Fruits (including 2 additional phytoestrogen-rich items, i.e. sesame/flaxseeds, and sunflower-/pumpkin-seeds) | | |
| 5. Dairy products | | |
| Cereals and cereal products | | |
| *6. Pasta, rice and other grain* | | |
| *7. Bread* | | |
| *8. Other cereals and cereal products* | | |
| Meat and meat products | | |
| *9. Red meat* | | |
| *10. Poultry* | | |
| *11. Processed meat* | | |
| 12. Fish and shellfish | | |
| 13. Eggs and egg products | | |
| Added fats | | |
| *14. Vegetable oils* | | |
| *15. Butter* | | |
| *16. Margarine* | | |
| *17. Deep-frying fat* | | |
| 18. Sugar and confectionery | | |
| 19. Cakes | | |
| 20. Non-alcoholic beverages | | |
| Alcoholic beverages | | |
| *21. Wine* | | |
| *22. Other alcoholic beverages* | | |
| 23. Sauces and condiments | | |
| 24. Soups and bouillons | | |
| *25. Soy products (including 4 additional phytoestrogen-rich items, i.e. soy milk, soy beans, tofu, soy spread)* | | |

Legenda: The 12 subgroups are in italics.
