# Supplementary material for: Dietary patterns and survival in German postmenopausal breast cancer survivors
Source: Br J Cancer. 2012 Nov 20;108(1):188–92. doi: 10.1038/bjc.2012.521 (PMC3553521; doi:10.1038/bjc.2012.521)
Supplement: Supplementary Table 1 [file bjc2012521x2.doc]

**Supplementary Table 1.** Baseline Characteristics of 2,522 Postmenopausal Breast Cancer Patients in the MARIE Study According to the Lowest and Highest Quartiles of the ‘Healthy’ and the ‘Unhealthy’ Dietary Pattern, Germany, 2001-2005

|  | **‘Healthy’ Dietary Pattern** | | | | |  | **‘Unhealthy’ Dietary Pattern** | | | | |
| --- | --- | --- | --- | --- | --- | --- | --- | --- | --- | --- | --- |
|  | **Quartile 1** | | | **Quartile 4** | |  | **Quartile 1** | | | **Quartile 4** | |
| No. of patients | 631 | | | 630 | |  | 631 | | | 630 | |
| Mean age at diagnosis, years^a^ | 63.8 | | (5.6) | 61.9 | (5.3) |  | 62.0 | (5.4) | | 63.0 | (5.4) |
| Tumor size, n (%)^b^ |  | |  |  |  |  |  |  | |  |  |
| ≤2 cm | 329 | | (52.1) | 359 | (57.0) |  | 367 | (58.3) | | 331 | (52.5) |
| >2-≤5 cm | 230 | | (36.5) | 216 | (34.3) |  | 203 | (32.2) | | 227 | (36.0) |
| >5 cm | 20 | | (3.2) | 18 | (2.9) |  | 21 | (3.3) | | 26 | (4.1) |
| Growth into chest wall/skin | 28 | | (4.4) | 13 | (2.1) |  | 17 | (2.7) | | 19 | (3.0) |
| Neoadjuvant CT | 22 | | (3.5) | 23 | (3.7) |  | 22 | (3.5) | | 25 | (4.0) |
| Missing | 2 | | (0.3) | 1 | (0.2) |  | 1 | (0.2) | | 2 | (0.3) |
| Nodal status, n (%) |  | |  |  |  |  |  |  | |  |  |
| 0 | 399 | | (63.2) | 419 | (66.5) |  | 420 | (66.7) | | 405 | (64.3) |
| 1-3 | 150 | | (23.8) | 129 | (20.5) |  | 146 | (23.2) | | 135 | (21.4) |
| 4-9 | 37 | | (5.9) | 40 | (6.3) |  | 29 | (4.6) | | 41 | (6.5) |
| ≥10 | 21 | | (3.3) | 18 | (2.9) |  | 14 | (2.2) | | 23 | (3.7) |
| Neoadjuvant CT | 22 | | (3.5) | 23 | (3.7) |  | 22 | (3.5) | | 25 | (4.0) |
| Missing | 2 | | (0.3) | 1 | (0.2) |  | 0 | (0.0) | | 1 | (0.2) |
| Metastases, n (%) |  | |  |  |  |  |  |  | |  |  |
| No | 603 | | (95.6) | 614 | (97.5) |  | 611 | (97.0) | | 610 | (96.8) |
| Yes | 28 | | (4.4) | 14 | (2.2) |  | 19 | (3.0) | | 19 | (3.0) |
| Missing | 0 | | (0.0) | 2 | (0.3) |  | 1 | (0.2) | | 1 | (0.2) |
| Tumor grade, n (%) |  | |  |  |  |  |  |  | |  |  |
| Low + moderate | 433 | | (68.6) | 436 | (69.2) |  | 456 | (72.4) | | 422 | (67.0) |
| High | 172 | | (27.3) | 170 | (27.0) |  | 151 | (24.0) | | 181 | (28.7) |
| Neoadjuvant CT | 22 | | (3.5) | 23 | (3.7) |  | 22 | (3.5) | | 25 | (4.0) |
| Missing | 4 | | (0.6) | 1 | (0.2) |  | 2 | (0.3) | | 2 | (0.3) |
| ERPR, n (%) |  | |  |  |  |  |  |  | |  |  |
| ER^+^PR^+^ | 397 | | (62.9) | 401 | (63.7) |  | 376 | (59.7) | | 406 | (64.4) |
| ER^+^PR^-^/ER^-^PR^+^ | 111 | | (17.6) | 104 | (16.5) |  | 121 | (19.2) | | 101 | (16.0) |
| ER^-^PR^-^ | 100 | | (15.8) | 102 | (16.2) |  | 111 | (17.6) | | 98 | (15.6) |
| Neoadjuvant CT | 22 | | (3.5) | 23 | (3.7) |  | 22 | (3.5) | | 25 | (4.0) |
| Missing | 1 | | (0.2) | 0 | (0.0) |  | 1 | (0.2) | | 0 | (0.0) |
| HER2, n (%) |  | |  |  |  |  |  |  | |  |  |
| HER2^+^ | 127 | | (20.1) | 102 | (16.2) |  | 112 | (17.8) | | 104 | (16.5) |
| HER2^-^ | 417 | | (66.1) | 443 | (70.3) |  | 454 | (72.1) | | 442 | (70.2) |
| Neoadjuvant CT | 22 | | (3.5) | 23 | (3.7) |  | 22 | (3.5) | | 25 | (4.0) |
| Missing | 65 | | (10.3) | 62 | (9.8) |  | 43 | (6.8) | | 59 | (9.4) |
| Type of surgery, n (%) |  | |  |  |  |  |  |  | |  |  |
| Ablation | 209 | | (33.1) | 192 | (30.5) |  | 181 | (28.7) | | 227 | (36.0) |
| BCT | 417 | | (66.1) | 431 | (68.4) |  | 444 | (70.5) | | 400 | (63.5) |
| Missing | 5 | | (0.8) | 7 | (1.1) |  | 6 | (1.0) | | 3 | (0.5) |
| Chemotherapy, n (%) |  | |  |  |  |  |  |  | |  |  |
| No | 327 | | (51.8) | 297 | (47.1) |  | 332 | (52.7) | | 295 | (46.8) |
| Yes | 301 | | (47.7) | 324 | (51.4) |  | 295 | (46.8) | | 327 | (51.9) |
| Missing | 3 | | (0.5) | 9 | (1.4) |  | 4 | (0.6) | | 8 | (1.3) |
| Radiotherapy, n (%) |  | |  |  |  |  |  |  | |  |  |
| No | 145 | | (23.0) | 125 | (19.8) |  | 122 | (19.4) | | 143 | (22.7) |
| Yes | 481 | | (76.2) | 498 | (79.0) |  | 505 | (80.2) | | 481 | (76.3) |
| Missing | 5 | | (0.8) | 7 | (1.1) |  | 4 | (0.6) | | 6 | (1.0) |
| Hormonal therapy, n (%) |  | |  |  |  |  |  |  | |  |  |
| No | 103 | | (16.3) | 103 | (16.3) |  | 110 | (17.5) | | 95 | (15.1) |
| Yes | 499 | | (79.1) | 500 | (79.4) |  | 496 | (78.7) | | 503 | (79.8) |
| Missing | 29 | | (4.6) | 27 | (4.3) |  | 25 | (4.0) | | 32 | (5.1) |
| Diabetes, n (%) |  | |  |  |  |  |  |  | |  |  |
| No | 580 | | (91.9) | 575 | (91.3) |  | 600 | (95.2) | | 555 | (88.1) |
| Yes | 49 | | (7.8) | 54 | (8.6) |  | 31 | (4.9) | | 73 | (11.6) |
| Missing | 2 | | (0.3) | 1 | (0.2) |  | 0 | (0.0) | | 2 | (0.3) |
| Cardiovascular disease, n (%) |  | |  |  |  |  |  |  | |  |  |
| No | 289 | | (45.8) | 318 | (50.5) |  | 350 | (55.6) | | 271 | (43.0) |
| Yes | 342 | | (54.2) | 312 | (49.5) |  | 281 | (44.6) | | 359 | (57.0) |
| Mode of detection, n (%) |  | |  |  |  |  |  |  | |  |  |
| Self-detected | 358 | | (56.7) | 342 | (54.3) |  | 329 | (52.2) | | 343 | (54.4) |
| Physician-detected | 270 | | (42.8) | 283 | (44.9) |  | 299 | (47.5) | | 286 | (45.4) |
| Missing | 3 | | (0.5) | 5 | (0.8) |  | 3 | (0.5) | | 1 | (0.2) |
| HRT use at diagnosis, n (%) |  | |  |  |  |  |  |  | |  |  |
| Never, past | 344 | | (54.5) | 337 | (53.5) |  | 314 | (49.8) | | 330 | (52.4) |
| Current | 284 | | (45.0) | 288 | (45.7) |  | 316 | (50.2) | | 293 | (46.5) |
| Missing | 3 | | (0.5) | 5 | (0.8) |  | 1 | (0.2) | | 7 | (1.1) |
| Smoking status, n (%) |  | |  |  |  |  |  |  | |  |  |
| Never | 353 | | (55.9) | 315 | (50.0) |  | 307 | (48.7) | | 369 | (58.6) |
| Past | 143 | | (22.7) | 207 | (32.9) |  | 198 | (31.4) | | 147 | (23.3) |
| Current | 135 | | (21.4) | 108 | (17.1) |  | 126 | (20.0) | | 114 | (18.1) |
| Adult BMI, n (%) |  | |  |  |  |  |  |  | |  |  |
| <18.5 kg/m^2^ | 16 | | (2.5) | 16 | (2.5) |  | 23 | (3.7) | | 17 | (2.7) |
| 18.5-25 kg/m^2^ | 470 | | (74.5) | 455 | (72.2) |  | 505 | (80.0) | | 426 | (67.6) |
| 25-30 kg/m^2^ | 126 | | (20.0) | 131 | (20.8) |  | 93 | (14.7) | | 152 | (24.1) |
| ≥30 kg/m^2^ | 19 | | (3.0) | 28 | (4.4) |  | 10 | (1.6) | | 35 | (5.6) |
| Leisure time PA since age 50, n (%) | | |  |  |  |  | |  |  |  |  |
| <28 METh/wk | | 194 | (30.7) | 160 | (25.4) |  | 157 | (24.9) | | 188 | (29.8) |
| ≥28 METh/wk | | 435 | (68.9) | 462 | (73.3) |  | 465 | (73.8) | | 437 | (69.4) |
| Missing | | 2 | (0.3) | 8 | (1.3) |  | 9 | (1.4) | | 5 | (0.8) |
| Occupation, n (%) | |  |  |  |  |  |  |  | |  |  |
| Low | | 275 | (43.6) | 192 | (30.5) |  | 173 | (27.5) | | 278 | (44.1) |
| Medium | | 218 | (34.5) | 270 | (42.9) |  | 263 | (41.7) | | 213 | (33.8) |
| High | | 135 | (21.4) | 167 | (26.5) |  | 193 | (30.6) | | 136 | (21.6) |
| Missing | | 3 | (0.5) | 1 | (0.2) |  | 2 | (0.3) | | 3 | (0.5) |
| Education, n (%) | |  |  |  |  |  |  |  | |  |  |
| Low | | 426 | (67.5) | 321 | (51.0) |  | 287 | (45.6) | | 417 | (66.2) |
| Medium | | 143 | (22.7) | 188 | (29.8) |  | 208 | (33.0) | | 146 | (23.2) |
| High | | 62 | (9.8) | 121 | (19.2) |  | 135 | (21.4) | | 67 | (10.6) |
| Missing | | 0 | (0.0) | 0 | (0.0) |  | 1 | (0.2) | | 0 | (0.0) |

Abbreviations: BCT, breast conserving therapy; BMI, body mass index; CT, chemotherapy; ERPR, estrogen receptor / progesterone receptor; HER2, human epidermal growth factor receptor 2; HRT, hormone replacement therapy; MET, metabolic equivalent value; PA, physical activity.

^a^ Number in parentheses, standard deviation.

^b^ Percentages do not always add up to 100 due to rounding.
