# Supplementary material for: Dietary patterns and survival in German postmenopausal breast cancer survivors
Source: Br J Cancer. 2012 Nov 20;108(1):188–92. doi: 10.1038/bjc.2012.521 (PMC3553521; doi:10.1038/bjc.2012.521)
Supplement: Supplementary Table 2 [file bjc2012521x3.doc]

**Supplementary Table 2.** Hazard Ratios of Overall Mortality, Breast Cancer-Specific Mortality, and Other Mortality Among Stage I-IIIa Breast Cancer Patients According to Quartiles of Dietary Patterns in the MARIE Study, Germany, 2001-2009

|  |  | **Overall mortality** | | |  | **Breast cancer mortality** | | |  | **Other mortality** | | |
| --- | --- | --- | --- | --- | --- | --- | --- | --- | --- | --- | --- | --- |
| **Quartiles of dietary pattern** | **No. of Subjects** | **No. of**  **Deaths** | **HR** | **95% CI** |  | **No. of**  **Deaths** | **HR** | **95% CI** |  | **No. of Deaths** | **HR** | **95% CI** |
| ‘Healthy’ pattern | | | | | | | | | | | | |
| Model 1^a^ |  |  |  |  |  |  |  |  |  |  |  |  |
| Q1 | 552 | 62 | 1.00 |  |  | 37 | 1.00 |  |  | 25 | 1.00 |  |
| Q2 | 567 | 50 | 0.90 | 0.61, 1.31 |  | 35 | 0.99 | 0.62, 1.59 |  | 15 | 0.75 | 0.39, 1.44 |
| Q3 | 565 | 50 | 0.84 | 0.57, 1.24 |  | 37 | 0.98 | 0.61, 1.58 |  | 13 | 0.61 | 0.31, 1.22 |
| Q4 | 572 | 44 | 0.76 | 0.51, 1.12 |  | 29 | 0.80 | 0.49, 1.31 |  | 15 | 0.70 | 0.36, 1.35 |
| *P*-trend |  |  | 0.04 | |  |  | 0.10 | |  |  | 0.18 | |
| Model 2^b^ |  |  |  |  |  |  |  |  |  |  |  |  |
| Q1 | 540 | 59 | 1.00 |  |  | 35 | 1.00 |  |  | 24 | 1.00 |  |
| Q2 | 555 | 45 | 0.91 | 0.61, 1.36 |  | 32 | 1.06 | 0.64, 1.75 |  | 13 | 0.71 | 0.35, 1.44 |
| Q3 | 555 | 49 | 0.86 | 0.57, 1.29 |  | 36 | 1.06 | 0.64, 1.77 |  | 13 | 0.62 | 0.30, 1.27 |
| Q4 | 554 | 42 | 0.74 | 0.47, 1.15 |  | 28 | 0.84 | 0.48, 1.47 |  | 14 | 0.63 | 0.30, 1.34 |
| *P*-trend |  |  | 0.02 | |  |  | 0.13 | |  |  | 0.14 | |
| ‘Unhealthy’ pattern | | | | | | | | | | | | |
| Model 1^a^ |  |  |  |  |  |  |  |  |  |  |  |  |
| Q1 | 574 | 48 | 1.00 |  |  | 38 | 1.00 |  |  | 10 | 1.00 |  |
| Q2 | 562 | 51 | 1.08 | 0.73, 1.62 |  | 32 | 0.85 | 0.52, 1.37 |  | 19 | 2.03 | 0.93, 4.43 |
| Q3 | 561 | 43 | 0.92 | 0.60, 1.39 |  | 29 | 0.80 | 0.49, 1.30 |  | 14 | 1.39 | 0.61, 3.15 |
| Q4 | 559 | 64 | 1.40 | 0.95, 2.05 |  | 39 | 1.03 | 0.65, 1.63 |  | 25 | 2.90 | 1.37, 6.16 |
| *P*-trend |  |  | 0.08 | |  |  | 0.76 | |  |  | 0.007 | |
| Model 2^b^ |  |  |  |  |  |  |  |  |  |  |  |  |
| Q1 | 563 | 46 | 1.00 |  |  | 37 | 1.00 |  |  | 9 | 1.00 |  |
| Q2 | 551 | 50 | 1.12 | 0.74, 1.70 |  | 31 | 0.86 | 0.52, 1.41 |  | 19 | 2.18 | 0.96, 4.96 |
| Q3 | 546 | 41 | 0.89 | 0.57, 1.39 |  | 28 | 0.73 | 0.43, 1.24 |  | 13 | 1.47 | 0.61, 3.56 |
| Q4 | 544 | 58 | 1.29 | 0.83, 2.01 |  | 35 | 0.91 | 0.54, 1.55 |  | 23 | 2.92 | 1.23, 6.94 |
| *P*-trend |  |  | 0.24 | |  |  | 0.81 | |  |  | 0.02 | |

Abbreviations: CI, confidence interval; ERPR, estrogen receptor / progesterone receptor; HR, hazard ratio; HRT, hormone replacement therapy; Q, quartile.

^a^ The model was stratified by age at diagnosis and study centre.

^b^ The model was stratified by age at diagnosis and study centre, and adjusted for tumor size, nodal status, metastases, tumor grade, ERPR status, radiotherapy, HRT use at diagnosis, mode of detection, total energy intake; the model for other mortality was additionally adjusted for cardiovascular disease; due to missing covariate values, 52 observations were not included in model 2. Other potentially confounding variables, as specified in Supplementary Table 1, were not statistically significant and did not change the risk estimates by ≥10% when tested in the model and were therefore not included in the final model.
